# Applying standardized drug terminologies to observational healthcare databases: a case study on opioid exposure

**DOI:** 10.1007/s10742-012-0102-1

**Published:** 2012-10-27

**Authors:** Frank J. DeFalco, Patrick B. Ryan, M. Soledad Cepeda

**Affiliations:** Janssen Pharmaceutical Research & Development, L.L.C. 920 Route 202, Raritan, NJ 08869 USA

**Keywords:** Observational databases, Classification systems, Coding standards, Drug exposures, OMOP

## Abstract

Observational healthcare databases represent a valuable resource for health economics, outcomes research, quality of care, drug safety, epidemiology and comparative effectiveness research. The methods used to identify a population for study in an observational healthcare database with the desired drug exposures of interest are complex and not consistent nor apparent in the published literature. Our research evaluates three drug classification systems and their impact on prevalence in the analysis of observational healthcare databases using opioids as a case in point. The standard terminologies compiled in the Observational Medical Outcomes Partnership’s Common Data Model vocabulary were used to facilitate the identification of populations with opioid exposures. This study analyzed three distinct observational healthcare databases and identified patients with at least one exposure to an opioid as defined by drug codes derived through the application of three classification systems. Opioid code sets were created for each of the three classification systems and the number of identified codes was summarized. We estimated the prevalence of opioid exposure in three observational healthcare databases using the three defined code sets. In addition we compared the number of drug codes and distinct ingredients that were identified using these classification systems. We found substantial variation in the prevalence of opioid exposure identified using an individual classification system versus a composite method using multiple classification systems. To ensure transparent and reproducible research publications should include a description of the process used to develop code sets and the complete code set used in studies.

## Introduction

### Background

Opioids are strong analgesics which are increasingly used for the treatment of chronic malignant and nonmalignant pain (Ballantyne and Mao 2003; Sullivan et al. 2008). Systematic reviews of randomized controlled trials have confirmed their short-term efficacy for the treatment of neuropathic pain, back pain, osteoarthritis, cancer pain, and fibromyalgia (Cepeda et al. 2007; Deshpande et al. 2007; Eisenberg et al. 2006; Furlan et al. 2006; Martell et al. 2007; Noble et al. 2008). However, these trials have limited follow-up periods (around 16 weeks) (Deshpande et al. 2007; Furlan et al. 2006; Noble et al. 2008) and in the trials with longer follow-up periods, the lack of generalizability of the findings has been identified as a serious shortcoming (Deshpande et al. 2007). Observational healthcare databases provide an opportunity to assess their long term safety in a population based setting.

In this research we explore the question of how opioid exposures can be identified in observational healthcare databases through the use of standard vocabularies and classification systems.

Although most observational healthcare databases capture individual patient drug exposures, there is no single, standard drug coding scheme. In general, finding a comprehensive and accurate list of drug codes for these studies is cumbersome and time consuming. Code sets can be inconsistent across investigators as it requires manual review of code lists, often generated through a simple text search and unique to a specific database. Code set development is susceptible to multiple forms of errors including the omission of relevant codes and inadvertent code inclusion.

In U.S. based databases, commonly used coding schemes include the National Drug Code (NDC) (National Drug Code Directory 2011), Generic Product Identifier (GPI) (Master Drug Data Base v2.5 (MDDB^®^) 2011) or Veterans Affairs National Drug File (NDF) (National Formulary 2011) while outside the U.S. different coding schemes will be found. In addition drug exposures are captured as procedural administrations and represented in adjacent coding schemes (i.e., Healthcare Common Procedure Coding System (HCPCS) (HCPCS General Information 2011)).

Even after a single database and coding terminology are selected for study, analysis is further complicated by the process for selecting the proper set of codes as most coding schemes lack an obvious biologically or ingredient-based organizational structure. In these cases a classification system may be selected and applied to the underlying coding scheme in order to identify a particular class of drug. The National Library of Medicine provides RxNorm (An Overview to RxNorm 2011) as a standardized nomenclature for clinical drugs that provides classifications of branded products and generic ingredients. Additionally there are multiple classification systems available including the First DataBank Enhanced Therapeutic Classification (ETC.) system (Enhanced Therapeutic Classification System 2011), World Health Organization (WHO) Anatomical Therapeutic Chemical (ATC) classification system (WHOCC-Structure and principles 2011), and Veterans Affairs (VA) National Drug File Reference Terminology (NDF-RT) (National Drug File-Reference Terminology (NDF-RT) 2011) and each varies in content and structure.

In an effort to address the challenge of multiple coding systems and terminologies, the Observational Medical Outcomes Partnership (OMOP) (Stang et al. 2010) compiled multiple standardized terminologies and classification systems into an interrelated vocabulary. This vocabulary relies on existing standards and mappings, and leverages work within the Unified Medical Language System’s Metathesaurus (UMLS-Metathesaurus 2011). This study evaluates the use of the OMOP vocabulary in a network of disparate observational databases and explores the ability of its multiple standardized terminologies and classification systems to define an appropriate pool of codes for opioid exposure.

## Materials and methods

Standard vocabularies, classification systems and their relationships were derived from the OMOP’s Standard Terminologies [(OMOP Standard Terminologies 2011); this reference contains the complete set of standard terminologies]. Based on the expertise of our research team, oxycodone was used as a seed ingredient to define an opioid drug grouping in each drug classification system. We identified the point at which oxycodone was categorized within each hierarchy and selected the highest-level drug class that subsumed oxycodone while still being inclusive of other opioid-related drugs. The classes identified through this process were ‘Analgesic-Narcotic’ in ETC., ‘Opioids’ in ATC, and ‘Opioid Agonist’ in NDF-RT. Iterative exploration of ingredients subsumed within these classes was used to exclude other potential classes. Source codes were identified as all codes mapped to any descendent concept within the identified drug class. The source codes identified from each classification system were compared to identify overlap across the three systems. The string terms for all opioid ingredients identified by the three systems (e.g. ‘oxycodone’, ‘hydrocodone’, ‘codeine’) were used in lexical searches amongst all NDC descriptions for codes not previously classified as ‘opioids’ to identify any potentially unmapped source codes. The prevalence of opioid exposure was estimated for each definition based on occurrence of at least one coded record as either an 11 digit NDC code from a pharmacy dispensing record or a HCPCS from a procedural administration.

While the focus of this paper is the exploration of classification system variation as it relates to opioids additional high level analysis was performed to ensure this was not simply an issue in one therapeutic category. The high level analysis was repeated for NSAIDs, Antidiabetics and Antidepressants and we found the variation also occurs in these other therapeutic areas. While we do not discuss these results further we have included the results for review (see Table 3).

To ensure that there was no substantial impact of the addition or removal of pharmacologic agents over time additional data has been provided which stratify the prevalence over time using each of the three individual classification systems (see Table 2).

Three observational healthcare databases were included in this study; the MarketScan Commercial Claims and Encounters (CCAE), Medicare Supplemental (MDCR) databases, MarketScan Medicaid (MDCD) database, and OptumInsight Clinformatics (OPTUM) database.

The MarketScan Commercial Claims and Encounters Database consists of employer and health plan sourced data for several million individuals containing medical and drug data linked to outpatient prescription drug claims and person-level enrollment information. Similar data are also available for the subset of employee retirees who have supplemental Medicare coverage (MDCR) (David et al. 2008). The MarketScan Medicaid Database captures similar data for Medicaid enrollees in several states (David et al. 2008). Results from the CCAE and MDCR databases were combined as the patient records can be continuous across the two databases (CCAEMDCR). The version of the CCAEMDCR database used in this study contained data from 2000 to 2009. The version of the MDCD database used in this study contained data from 2006 to 2008.

The OptumInisght (OPTUM) Clinformatics database contains patient-level data inclusive of administrative data, pharmacy claims data, physician and facility claims data, and lab test results from enrollees in managed care plans administered by United Health Group (i3 InVision Data Mart 2010). The version of the OPTUM database used in this study contained data from 2005 to 2010.

While the observational healthcare databases used in this study are only available under licenses from their respective organizations, the OMOP vocabulary is publically available allowing further investigation of our results against other available data sources.

An opioid related literature review was conducted to assess the proportion of papers that explicitly articulate the code set used or a description of the process used to develop the code set.

## Results

### Composite code mappings

Basic information for each database was captured to provide a summary of the number of prescription drug claim records and unique drug codes represented. We found that between 55.8 and 69.2 % of 11 digit NDC codes in the observational databases were successfully mapped to the concepts represented in the OMOP standard vocabulary. The set of mapped codes accounted for between 93.8 and 95.1 % of the total prescription drug claim records found in the observational databases (see Table 1).

**Table 1:** Composite code mappings across three observational healthcare databases

	Observational healthcare databases		
	CCAEMDCR	MDCD	OPTUM
No. distinct drug codes (11 digit NDC)	133,117	47,605	67,031
No. drug records	2,605,047,390	133,879,982	691,892,761
No. mapped codes	74,288	32,977	42,439
No. records covered by mapping	2,479,374,599	126,094,396	649,029,503
% of codes mapped	55.8	69.3	63.3
% of records covered by mapping	95.2	94.2	93.8

### Classification systems

**Fig. 1:**
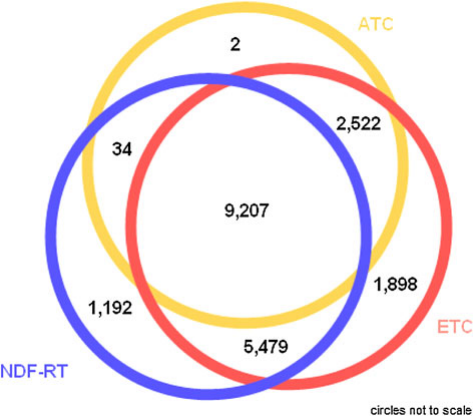
Overlap in coverage of ‘opioid’ NDC drug codes by classification system

A substantial overlap between the opioid definitions based on the ATC, NDF-RT and ETC. classification systems was found by comparing the 11 digit NDC codes they identified. 9,207 NDCs were captured by all of the 3 classification systems. (see Fig. 1). Each of the drug classification systems also yielded NDCs that were not found by any of the other two systems; 1,192 NDC codes were uniquely identified using NDF-RT, 1,898 codes were uniquely identified using ETC., and 2 codes were uniquely identified using ATC (see Table 3). The full set of NDC codes identified by all classification systems is provided in Appendix 1 which is available upon request due to its large size.

The prevalence of opioid usage was estimated across each of the observational healthcare database using the three classification systems. If ‘opioid’ were defined only by ‘Opioids’ class in ATC, the observed prevalence in CCAEMDCR was 19.2 %. Defining ‘opioids’ using the ETC. ‘Narcotic analgesic’ class yielded a prevalence of 31.6 % while defining ‘opioids’ as all products based on the ‘Opioid agonist’ mechanism of action in NDF-RT produced a CCAEMDCR prevalence of 28.8 %. Using a composite set of 11 digit NDCs based on all three classification systems produced a prevalence of 33.1 %. (see Table 2).

**Table 2:** Opioid prevalence by classification system and observational healthcare database

Classification system	Index year	CCAEMDCR			MDCD			OPTUM		
		No. of persons with record	% of database	No. of records	No. of persons with record	% of database	No. of records	No. of persons with record	% of database	No. of records
Combined	2006	5,153,607	20.8	15,358,270	1,048,333	16.6	3,771,726	2,506,491	17.5	6,876,745
Combined	2007	5,493,069	20.7	16,819,905	606,704	14.6	2,429,126	2,550,448	17.4	7,102,600
Combined	2008	6,630,102	21.4	20,508,096	751,978	14.1	3,083,632	2,668,787	18.3	7,617,796
Combined	2009	7,590,919	21.7	23,452,003	–	0.0	–	2,571,817	18.5	7,435,742
Combined	2010	6,562,335	20.9	20,974,763	–	0.0	–	2,293,469	17.1	6,563,863
Combined	All	27,994,842	33.1	153,999,069	1,845,054	22.5	9,284,484	9,619,127	28.9	42,327,698
ATC	2006	2,603,155	10.5	7,075,478	670,360	10.6	1,968,729	1,205,085	8.4	3,102,653
ATC	2007	2,792,815	10.5	7,810,406	404,263	9.8	1,359,196	1,213,460	8.3	3,185,457
ATC	2008	3,337,609	10.8	9,465,529	503,856	9.5	1,725,418	1,263,062	8.6	3,431,591
ATC	2009	3,800,786	10.8	10,811,288	–	0.0	–	1,197,241	8.6	3,342,477
ATC	2010	3,372,605	10.7	9,974,062	–	0.0	–	1,069,493	8.0	2,974,644
ATC	All	16,244,578	19.2	71,638,220	1,258,848	15.3	5,053,343	5,079,401	15.3	18,985,594
ETC	2006	4,729,084	19.1	14,383,571	970,008	15.3	3,626,682	2,302,799	16.1	6,459,326
ETC	2007	5,052,707	19.1	15,796,840	571,523	13.8	2,351,409	2,343,945	16.0	6,672,976
ETC	2008	6,242,300	20.2	19,582,964	734,031	13.8	3,035,528	2,507,332	17.2	7,267,156
ETC	2009	3,800,786	20.7	22,608,707	–	0.0	–	2,443,146	17.6	7,149,797
ETC	2010	6,322,111	20.1	20,359,814	–	0.0	–	2,197,957	16.4	6,344,504
ETC	All	26,770,347	31.6	146,188,729	1,751,389	21.3	9,013,619	9,167,882	27.5	40,342,385
NDFRT	2006	4,465,908	18.0	13,752,756	867,842	13.7	3,351,981	2,155,453	15.0	6,150,699
NDFRT	2007	4,808,840	18.2	15,211,258	501,420	12.1	2,163,320	2,217,178	15.1	6,410,882
NDFRT	2008	5,663,678	18.3	18,315,335	605,836	11.4	2,723,708	2,269,416	15.5	6,798,260
NDFRT	2009	6,389,135	18.2	20,806,058	–	0.0	–	2,163,457	15.6	6,611,540
NDFRT	2010	5,643,499	17.9	18,877,278	–	0.0	–	1,957,487	14.6	5,878,918
NDFRT	All	24,335,198	28.8	136,254,056	1,511,999	18.4	8,239,009	8,361,068	25.1	37,837,561

### Classification hierarchies

Table 3 highlights the vocabulary classification of opioid-related ingredients identified by the three classification systems. NDF-RT has a classification based on mechanism of action. In this study we used drugs classified as ‘Opioid agonists’, however several of the qualifying drugs, such as buprenorphine are also classified as ‘opioid antagonists’. Alfentanil and codeine were not classified as opioid agonists, but instead are represented with the ‘opioid receptor interactions’ mechanism of action. The ATC classification system contains a high-level class for ‘opioids’, which is further segregated by ‘natural opioid alkaloids’ and various derivatives, including phenylpiperidine, benzomorphan, oripavine, and morphinan. Alfentanil, remifentanil, and sufentanil are classified elsewhere under ‘Anesthetics, general’ as ‘opioid anesthetics’. Surprisingly, hydrocodone is not classified in the ‘opioid’ class, but instead placed under the classes of cough suppressants (including ‘opium derivatives and expectorants’ and ‘opium alkaloids and derivatives) and other analgesics under ‘anilides’. Within the ETC’s hierarchy, most active ingredients of interest are subsumed within the class of ‘Narcotic analgesic’, which was further stratified by combination ingredient. There is no concept for the term ‘opioid’ within the ETC. classification system. Notably, opium is classified elsewhere as an antidiarrheal and GI antispasmodic combination, while remifentanil and sufentanil are classified as ‘generic anesthetic adjuncts–narcotic’.

**Table 3:** Identification of related 11 digit NDC codes by drug class and vocabulary

Drug class	Vocabulary	System grouping	Ingredients	Clinical drugs	NDC codes	Unique codes
Opioid	ATC	Opioids	23	1,122	11,765	2
Opioid	ETC.	Analgesics–narcotic	20	1,808	19,106	333
Opioid	NDFRT	Opioid agonists	22	1,813	15,912	1,087
Opioid	VA	Opioid analgesics	24	1,750	17,113	450
NSAID	ATC	Antiinflam and antirheumatic products, non-steroids	52	1,109	18,519	374
NSAID	ETC.	NSAID analgesics	23	970	18,160	–
NSAID	NDFRT	NSAID analgesics	23	970	18,160	–
NSAID	VA	Nonsalicylate NSAIDs, antirheumatic	24	926	18,290	195
Antidiabetic	ATC	Drugs used in diabetes	53	483	7,475	47
Antidiabetic	ETC.	Oral antidiabetic agents	19	309	7,197	77
Antidiabetic	NDFRT	Insulin receptor agonists	42	445	7,114	14
Antidiabetic	VA	Oral hypoglycemic agents	18	273	6,965	–
Antidepressant	ATC	Antidepressants	47	665	17,542	246
Antidepressant	ETC.	Antidepressants	29	608	17,419	3
Antidepressant	NDFRT	Serotonin uptake inhibitors, norepinephrine uptake inhibitors, dopamine uptake inhibitors	40	1,030	20,670	4,406
Antidepressant	VA	Antidepressants	29	604	17,114	–

## Discussion

Standard drug vocabularies have utility in many applications. They provide a useful tool for comparing the prevalence of drugs across disparate data sources, assist in the identification of drugs within a class, and can help define a comparator population based on treatments for the same indication. Vocabularies can also help identify codes for exposures that appear as drug administration procedures as well as all combination drugs that include a specific active ingredient.

A relatively low percentage of all distinct drug codes found in the observational healthcare databases can be mapped to the standard drug vocabularies used in this study (55.8–69.2 %). The codes that do not map appear to be due to erroneous coding in the source data, incomplete mappings, and limitations of the target vocabularies. Despite the low percentage of distinct code mappings we found that a vast majority of data records were successfully mapped to the used vocabularies (93.8–95.2 %). While further work can potentially enhance the quality and completeness of code mappings, we believe that the law of diminishing returns will quickly reduce the amount of additional information captured through a more extensive mapping. The biggest benefit of applying drug classification standards would be in eliminating erroneous data by ensuring that all drug codes match standard vocabularies.

It is clear through this opioid example that there is no superior classification system and that there is substantial value in using multiple drug classification systems concurrently to reduce the risk of under-ascertainment of exposure. For example, restricting to only the ATC system would lead to the exclusion of hydrocodone—one of the most commonly prescribed opioids—as well as buprenorphine and methadone that, although used for the treatment of opioid dependence, are also used for the treatment of pain. Restricting to only the NDF-RT system would lead to the exclusion of tapentadol, a recently approved opioid. Additionally restricting to only the NDF-RT system would lead to the exclusion of alfentanil, but to the inclusion of other similar opioids with only intravenous formulations such as sufentanil.

Each classification system simply reflects a different perspective for organizing clinical concepts and the most value can be realized by leveraging multiple perspectives, which would lead to a more complete representation. While ETC. had the highest coverage of the opioid NDCs, the high level analysis of the three other therapeutic areas showed that no single classification system consistently exhibited this capability.

In our opinion, inclusion of drug code sets and a description of the process used to develop code sets in publications would provide a significant value for the healthcare research community. This level of detail is generally lacking in current literature. A literature search was performed to determine how often authors reported the complete set of drug codes used in observational database studies. The intent of this review was to illustrate the potential for variation while attempting to reproduce other study results. We limited the search to studies published in English and used the Medical Subject Headings terms: “epidemiologic studies”, “case–control studies”, “cohort studies”, and “follow-up studies” combined with “Analgesics, Opioids” and the key word: “database”. Out of the 23 studies (Cepeda et al. 2010; Chen et al. 2010; Franklin et al. 2008; Gallagher et al. 2009; Gasse et al. 2000; Goettsch et al. 2007; Gross et al. 2009; Iyer et al. 2010; Jick et al. 1998; Kwong et al. 2010; Massey et al. 2005; Parente et al. 2004; Pradel et al. 2004; Sittl et al. 2005; Skurtveit et al. 2010; Skurtveit et al. 2008; Sullivan et al. 2010; Victor et al. 2009; Voaklander et al. 2008; Von Korff et al. 2011; White et al. 2009; Ytterberg et al. 1998; Zorowitz et al. 2005) we identified as observational studies using electronic medical record or claims databases, only three reported the drug codes used. Notably, the authors of these manuscripts were the same and the codes provided were identical. Understanding the process used to develop code sets provides a way for researchers to understand the decisions made in the framing of the research question and in turn, determine possible implications for replication of the results.

Code sets can be very large making peer reviewed publications a less than ideal location to present them, however, they could be made available as an online supplement. Perhaps a public library of code sets with appropriate definitions could be made available through the National Library of Medicine or some other curated source.

## Limitations

This is a single study of a single drug class and more research of additional drug classes would be required to further support our conclusions.

Even with shared code sets reproducing results could be challenging given the fact that observational healthcare databases have non-standard versions, formats, update frequencies and time frames across different organizations.
